# Dysbiosis and the gut–brain axis impairment in the pathophysiology of Alzheimer’s disease and related dementias: is ‘pathobiome’ an etiological element?

**DOI:** 10.1042/EBC20253055

**Published:** 2026-03-24

**Authors:** Gwoncheol Park, Paramita Chakrabarty, Philip A. Efron, Ravinder Nagpal

**Affiliations:** 1 The Gut Biome Lab, Department of Health, Nutrition, and Food Sciences, College of Education, Health, and Human Sciences, Florida State University, Tallahassee, FL 32306, U.S.A.; 2 Center for Translational Research in Neurodegenerative Disease, McKnight Brain Institute, Department of Neuroscience, University of Florida, Gainesville, FL 32610, U.S.A.; 3 Department of Surgery, Sepsis and Critical Illness Research Center, University of Florida College of Medicine, Gainesville, FL 32610, U.S.A.

**Keywords:** Alzheimer’s disease, cognitive impairment, dysbiosis, dementia, gut–brain axis, gut microbiome, neurodegeneration, neuroinflammation, sepsis

## Abstract

The gut microbiome plays a pivotal role in host metabolic, cardiovascular, and immune health. Increasing evidence also links it to aging-associated neurocognitive decline and neurodegenerative disorders, including Alzheimer’s disease (AD) and related dementias. While the precise mechanisms of the gut–microbiome–brain axis remain incompletely understood, recent findings challenge the traditional view of AD as a disease confined to the central nervous system. Aging-associated gut dysbiosis, marked by loss of beneficial microbes, expansion of opportunistic pathogens, and reduced microbial diversity, can compromise intestinal barrier integrity, leading to ‘leaky gut’ and increased translocation of microbial components or pathogens into the circulation. These elements may cross a weakened blood-brain barrier, triggering neuroinflammation, amyloid-beta accumulation, tau hyperphosphorylation, and neuronal injury. Such pathobiome-driven inflammatory cascades may initiate or accelerate AD pathology, shifting the etiological perspective beyond the amyloid and tau hypotheses toward systemic and peripheral contributors. Our work and others’ have identified distinct dysbiotic microbiome signatures in AD, supporting the possibility that AD pathogenesis may begin in the gut. Restoring microbial homeostasis through targeted interventions could attenuate neuroinflammatory and neurodegenerative processes, offering a novel preventive and therapeutic avenue. This emerging paradigm underscores the need for comprehensive, mechanistic, and longitudinal studies to define how aging-driven microbiome alterations influence the gut–brain axis and contribute to AD progression.

## Introduction

An increased predisposition to cognitive impairment and dementia is one of the critical outcomes of human aging. Alzheimer’s disease (AD), the most prevalent neurodegenerative disorder worldwide at present, is the leading cause of dementia, representing about 60%–70% of all cases [1]. AD is primarily a disease of the brain; however, studies over the past decade suggest that specific pathologic elements in the peripheral tissues and organs might also be involved. Alterations in the immune-inflammatory processes including aggravated inflammation and microglial activation in patients with mild cognitive impairment (MCI) and AD indicate the role for a cross-talk between the central and peripheral immune system [2,3]. However, the precise mechanisms and origins of these triggers remain largely unclear.

One emerging concept involves aging-associated dysbiosis, an imbalance in gut microbial communities that develops with age, which can impair epithelial barrier integrity, promote systemic inflammation, and alter immune homeostasis [4]. This state is often described within the framework of the pathobiome, in which disruption of a health-promoting and ecologically stable gut microbiome leads to dysbiosis characterized by reduced microbial diversity and altered metabolic function, thereby increasing susceptibility to pathogen invasion and overgrowth [5]. These microbiome changes may influence neuroinflammatory pathways, potentially accelerating neurodegenerative processes.

Evidence from other high-biomass microbial niches provides a precedent for this concept. For example, oral dysbiosis in periodontitis has been strongly associated with AD. The periodontal keystone pathogen *Porphyromonas gingivalis* has been detected in AD brains and is proposed to contribute to pathology through mechanisms including brain colonization, increased production of amyloid-β_1-42_, and tau cleavage mediated by its cysteine proteases, gingipains [6,7]. This example illustrates how chronic dysbiosis at a peripheral mucosal site can exert systemic and neuroinflammatory consequences.

Taken together, these observations support the hypothesis that gut dysbiosis, given its extensive microbial biomass, metabolic activity, and intimate immune interactions, may similarly act as an upstream modulator of neurodegeneration. Moreover, the repeated failure of numerous clinical trials aimed exclusively at amyloid and tau pathologies underlines the need to address this debilitating disorder from a different perspective. Accordingly, there is currently a striking research impulsion to obtain an all-inclusive understanding of potential mechanisms including the pathological/etiological and genetic factors, as well as the gut microbiome, which may contribute to disease onset. This encompasses AD and related dementias (ADRD), including frontotemporal degeneration, Lewy body dementia, vascular contributions to cognitive impairment and dementia, and multiple-etiology dementias, which share many cognitive and pathological features with AD and can be challenging to distinguish from it [8–10].

In recent years, multiple comprehensive reviews have summarized the involvement of the gut microbiome in ADRD, largely emphasizing gut–brain communication, microbial associations, and neuromodulatory metabolites [11–14]. These studies have been instrumental in establishing the relevance of the gut microbiome to neurodegeneration. Building on this growing body of work, accumulating evidence suggests that aging-associated dysbiosis may represent an important upstream contributor to peripheral immune activation and chronic inflammation relevant to ADRD pathophysiology. In light of limited success of therapeutic strategies targeting amyloid and tau alone, there is increasing interest in integrative models that consider peripheral and systemic factors in AD. Accordingly, this review aims to synthesize recent evidence linking dysbiotic changes, systemic inflammation, and neuroinflammatory mechanisms, while highlighting conceptual gaps and experimental challenges that must be addressed to clarify the role of gut dysbiosis in the etiology and progression of AD and related dementias.

## Aging-associated changes in the gut health: a foundation for systemic decline

Aging-related changes in intestinal health result from a combination of intrinsic (biological) and extrinsic (lifestyle) factors. Recent studies have begun to unravel the mechanistic underpinnings of how aging affects gut physiology. For instance, the accumulation of mitochondrial DNA mutations impairs blastocyst formation, contributing to physiological intestinal aging [15], while a decline in protein homeostasis within intestinal stem cells disrupts aggregate clearance and cell cycle progression, leading to age-related barrier dysfunction [16]. Additionally, aging is associated with reduced goblet cell numbers, resulting in a thinner mucus layer that leaves the epithelium more exposed to bacteria and vulnerable to microbial invasion [17,18]. Transcriptional and epigenetic changes in the aging gut have also been reported. One study highlighted age-associated epigenetic regulation affecting Group 3 innate lymphoid cells, which play a crucial role in intestinal immunity, thereby increasing susceptibility to bacterial and fungal infections [19]. Another study found age-related gene expression changes linked to stress and immune responses in the colon and mesenteric lymph nodes, changes that were closely tied to gut microbiome composition [20]. Notably, microbiome depletion in aged mice improved gut barrier integrity, while fecal microbiota transplantation from young to old mice significantly enhanced both barrier function and immune homeostasis—indicating that gut microbial profiles strongly influence, and are influenced by, age-associated physiological changes [4,20,21].

We previously proposed the term biome-aging to describe the transformation of the gut microbiome with age, emphasizing its central role in regulating the pace of biological aging [4]. The aging microbiome is typically characterized by reduced metabolic activity and diminished beneficial microbial interactions, which lead to increased systemic inflammation and downregulation of host pathways critical for maintaining intestinal barrier function [22]. For example, *Akkermansia muciniphila*, which tends to decline with age, has been closely linked to gut health and age-associated disorders in rodent studies [23–25]. Supplementation with *A. muciniphila* has been shown to prevent age-related thinning of the colonic mucus layer [24], and its derivatives, such as acetic acid and the protein Amuc_1409, contribute to gut health and healthy aging [23,25]. Furthermore, studies on the long-lived individuals have revealed specific associations between gut pathogens and systemic inflammatory markers (e.g., *Klebsiella pneumoniae* and liver disease markers), and long-lived individuals often retain youthful microbiome signatures, suggesting that the trajectory of gut microbiome aging may influence susceptibility to chronic diseases and the overall aging process. Consistent with this framework, a growing body of evidence demonstrates that dietary patterns, microbiome-targeted interventions (including probiotics, prebiotics, postbiotics, and fecal microbiota transplantation), and microbiome-derived metabolites can beneficially modulate gut barrier integrity, systemic inflammation, metabolic health, and neurocognitive function during aging. These observations position the gut microbiome as a modifiable and actionable determinant of healthy aging and longevity, with therapeutic potential to mitigate age-associated disorders, including neurodegenerative diseases. For a more comprehensive discussion of microbiome-based therapeutic strategies aimed at reversing or attenuating age-associated disorders, readers are referred to our recent review [4].

In summary, age-related intestinal physiological changes are intimately linked with biome-aging in a bidirectional manner. This interaction ultimately compromises gut integrity and drives chronic low-grade inflammation, laying the foundation for aging-associated diseases. However, these processes may be modifiable, or even reversible, through restoration of gut microbial homeostasis and targeted intervention to reset biome-aging.

## Gut dysbiosis and neuroinflammation: a pathogenic link to ADRD

**Figure 1:**
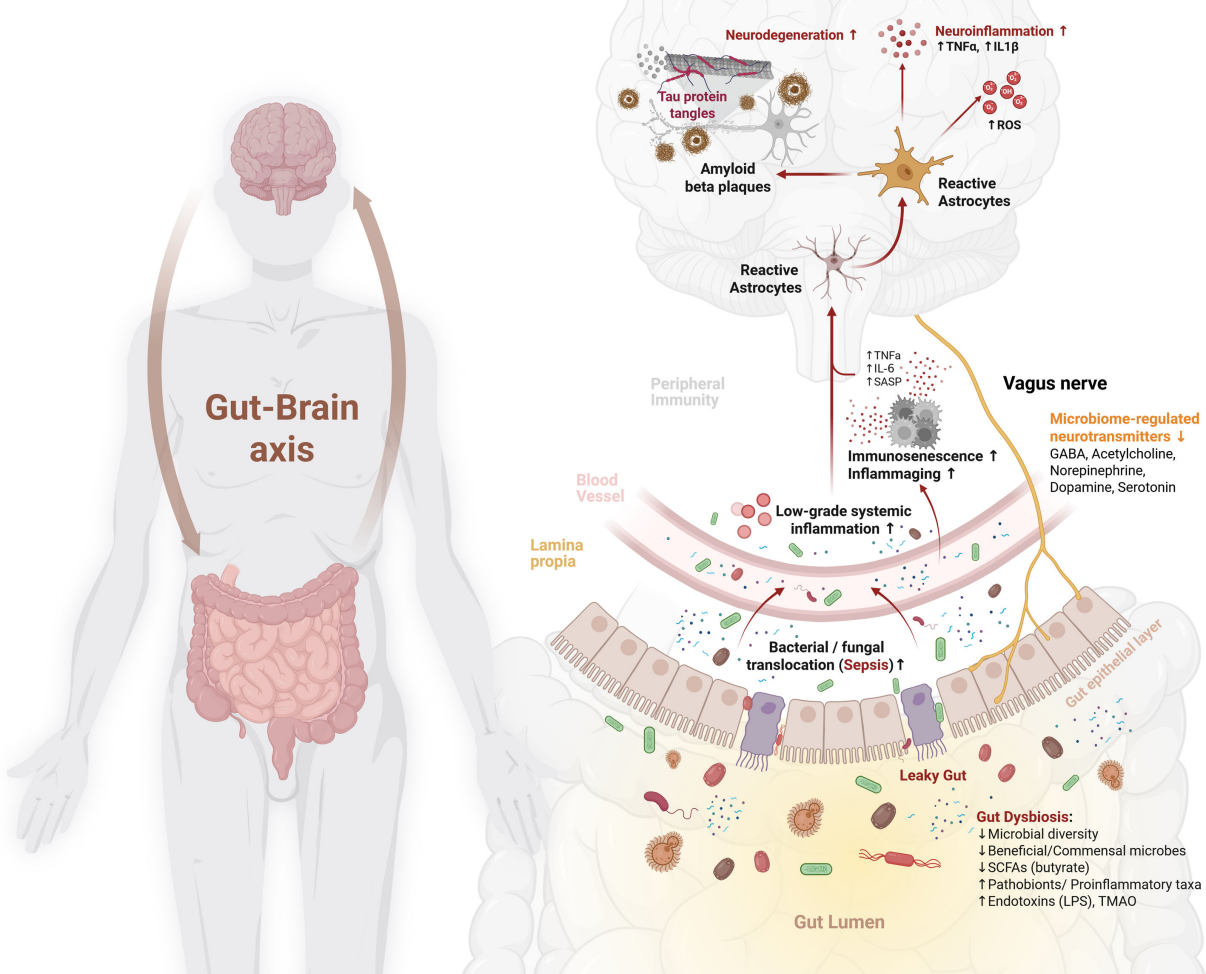
An outline of purported mechanisms implicating gut microbiome dysbiosis in the pathophysiology of Alzheimer’s disease and related dementias. Abnormal perturbations in the gut microbiome (‘gut dysbiosis’) due to aging, infections, drugs, dietary/lifestyle factors, etc., cause declined diversity and beneficial microbes and increased indigenous pathobionts and opportunistic pathogens in the gut, which trigger local inflammation and impaired gut epithelial membrane integrity (hyperpermeability; ‘leaky gut’) leading to gut-to-blood translocation of proinflammatory bacteria and toxins (sepsis) to the circulation thereby inciting systemic low-grade inflammation and impaired regulation of pathways associated with microbiome-associated neurotransmitters. This sepsis and systemic inflammation incite dysregulation/ impairment of the blood-brain barrier and/or vagus nerve elements, which instigate elevated neuroinflammation, oxidative stress, amyloid aggregation, tau hyperphosphorylation, and neurodegeneration, eventually leading to memory deficits, neurocognitive decline, and dementia and AD. SCFA: sort-chain fatty acids; LPS: lipopolysaccharides; TMAO: trimethylamine-N-oxide; GABA: gamma-amino butyric acid; ROS: reactive oxygen species; TNFa: tumor necrosis factor-alpha; SASP: senescence-associated secretory phenotype; IL: interleukin; Immunosenescence: the gradual decline of immune function with age; Inflammaging: chronic low-grade systemic inflammation associated with aging, further contribute to gut dysbiosis, systemic inflammation, and neurodegenerative processes.

One of the critical barriers in developing effective treatments against ADRD is the multi-factorial pathologies of various heterogeneous disorders that underlie cognitive decline [26]. Current evidence suggests that common neuropathological mechanisms across ADRD include amyloid-β aggregation and tau hyperphosphorylation, which are hallmark features of AD; α-synuclein accumulation, which is central to Lewy body dementia and Parkinson’s disease dementia; as well as oxidative stress, synaptic dysfunction, and neuroinflammation, which are shared across multiple dementia subtypes [27–29]. Although the relative contribution of each mechanism varies by disorder, these processes are highly interrelated [30]. For example, amyloid-β and tau pathology in AD can trigger microglial activation and oxidative stress, while α-synuclein aggregation in Lewy body dementia and Parkinson’s disease dementia similarly promotes neuroinflammation and neuronal injury [30,31]. In all cases, these interlinked mechanisms establish a feed-forward cycle that accelerates neurodegeneration and cognitive decline [32]. However, recent studies have attributed cognitive deficits to several non-brain disorders including insulin resistance, obesity, and chronic low-grade systemic inflammation [33–37], all of which also involve an abnormal gut microbiome (‘gut dysbiosis’) and impaired epithelial integrity thereby substantiating the role for the gut–microbiome–brain interface in the pathophysiology of cognitive decline and ADRD (Figure 1). Neuroinflammatory triggers can stem not only from local factors, e.g., amyloid buildup, but also from peripheral origins such as microglial stimulation caused by elevated pro-inflammatory factors including cytokines, reactive oxygen species, or nitric oxide, highlighting neuroinflammation, an inflammatory response within the central nervous system mediated primarily by microglia and astrocytes, as a process potentially driven or modulated by the gut microbiome in ADRD [38].

Gut microbiome has lately received remarkable attention due to emerging understanding of its impacts on neurological health. Gut microbiome produce several neurotransmitters including gamma-amino butyric acid, acetylcholine, dopamine, serotonin, toxins, and specific vitamins all of which are known to modulate the vagus nerve signaling [39–45]. Studies have also reported abnormal levels of several microbiome metabolites, e.g., lactate and ammonia, in patients with dementia [46,47]. Conversely, specific microbiome-derived compounds, i.e., lipopolysaccharides (LPS; an endotoxin), have also been shown in human neuronal cells *in vitro* to translocate from gut to brain via aging-associated leaky gut and weakened gut–blood–brain barrier (BBB) [48], thereby provoking neuropathological cascades such as elevated neuroinflammation, microglial activation, hippocampal amyloid accumulation, and tau hyperphosphorylation eventually resulting in neurocognitive impairment [48–52]. On the other hand, the absence of microbiome (germ-free animals) has been associated with impaired microglia, immune and amyloid processes [53–55], linking the gut microbiome with ADRD.

Recent clinical studies by us and others have revealed specific gut dysbiosis arrays characterized by reduced population of gut commensal/beneficial clades including *Bacteroides*, *Lactobacillus* and *Bifidobacterium* and a congruently increased abundance of *Proteobacteria* and several other opportunistic pathobionts, with cognitive impairment and AD [56–59], which have been associated with deteriorated levels of neurotransmitters including gamma-amino butyric acid, acetylcholine, or serotonin, leading to cognitive decline [60–62]. Studies have also reported higher levels of trimethylamine-N-oxide (TMAO) in the cerebrospinal fluid of AD patients, indicating the role of gut microbial metabolism in amyloid/tau pathology and neurodegeneration in particular context to vascular dementia [63–65]. Beyond AD, elevated TMAO has been linked to cardiovascular and metabolic conditions including heart disease, stroke, hypertension, and heart failure, which themselves are established risk factors for cognitive decline and dementia [66–68]. These observations suggest that TMAO may contribute to neurodegenerative processes both directly, via modulation of amyloid/tau pathology, and indirectly, through systemic vascular and inflammatory pathways associated with dementia risk. In addition, our recent clinical studies have reported that specific dietary manipulations may improve cerebrospinal markers of cognitive impairments and AD via modulating the gut microbiome [56,57,69]. Dietary-induced remodeling of the microbiome, including increased production of SCFAs, correlates negatively with AD biomarkers such as amyloid-β, supporting a mechanistic link between diet, microbial metabolism, and neuroprotection [57]. Notably, these observations of gut dysbiosis in patients with mild cognitive impairment, an early stage of AD, indicate that the elements of gut dysbiosis precede AD development and hence may even be involved in the etiology of ADRD. Several large-scale surveys and meta-analysis have also linked specific infectious agents, e.g., *Helicobacter pylori*, oro-intestinal pathogens, and human herpes viruses with the multi-factorial pathophysiology of ADRD [70–73], pointing towards an etiological role of gut pathobiome in ADRD. This is further corroborated by studies reporting elevated levels of Calprotectin, a marker of intestinal inflammation, in both stool and cerebrospinal fluid of AD patients, indicating a connection of gut inflammasome, a multiprotein complex that activates inflammatory responses, with amyloid pathology and neurocognitive impairment [74–76]. Accordingly, researchers have even proposed a new term ‘mapranosis’ to describe a process in which microbial components, including bacterial amyloids and other microbiota-derived factors, drive host protein misfolding in a prion-like manner and promote neuroinflammation [77]. In this framework, microbial amyloids can cross-seed aggregation of host amyloid proteins such as amyloidβ and α-synuclein, exacerbate neuroinflammatory signaling, and potentially contribute to the initiation and propagation of neurodegenerative pathology in ADRD [78].

Notably, comparable patterns have been observed in Lewy body diseases, where patients frequently experience gastrointestinal dysfunction, including constipation and intestinal bacterial overgrowth. These symptoms are regarded as some of the earliest prodromal features of Parkinson’s disease [79–81], suggesting that gastrointestinal disorders may increase the risk of Lewy body diseases or be closely associated with α-synuclein aggregation. Growing evidence indicates that α-synuclein, the major pathological hallmark of Lewy body diseases, forms pathological aggregates that cause neuronal damage and trigger clinical manifestations such as motor dysfunction and cognitive decline. Notably, α-synuclein may originate in the gut and subsequently spread to the central nervous system via prion-like cell-to-cell transmission, thereby facilitating further α-synuclein aggregation in the brain and initiating disease progression [82]. Colonic biopsies obtained from prodromal Lewy body disease cohorts, particularly patients with idiopathic REM sleep behavior disorder (RBD), have demonstrated the presence of α-synuclein, and gut microbes, including *Escherichia coli*, have been shown to promote α-synuclein-mediated neuroinflammation and motor deficits in rodent models [83–85]. Similar to the acceleration of amyloid-β plaque and tau tangle formation observed in AD as a consequence of dysbiosis, microbial imbalance and the resulting impairment of gut barrier integrity are now widely recognized as critical mechanisms driving Lewy body disease pathogenesis. These processes initiate a vicious cycle in which increased gut permeability, systemic inflammation, and α-synuclein aggregation perpetuate one another [86–89]. A systematic review by Tan et al*.* comparing 30 clinical studies demonstrated that an increase in the genus *Akkermansia* was the most consistently observed microbiome alteration in Parkinson’s disease [89]. In line with this finding, recent clinical observational studies have reported enrichment of *Escherichia* and *Akkermansia* in individuals with prodromal Parkinson’s disease or Parkinson’s disease accompanied by RBD, and these observations were further validated in independent cohorts, indicating common and reproducible gut microbiome alterations in patients with Lewy body diseases [90,91]. Similar microbial shifts have also been demonstrated in preclinical studies [92]. Additionally, a reduction in SCFA-producing bacteria has been widely reported across numerous recent clinical studies of Lewy body diseases [90,93–95]. Collectively, these pathobiotic alterations in the gut are thought to be associated with pathogenic biofilm formation and host mucin degradation, processes that exacerbate gut permeability and trigger inflammatory responses [90,93].

## Aging-induced dysbiosis, sepsis, and ADRD predisposition

Given that the gut microbiome serves as a critical line of defense against pathogenic infections, age-related loss of microbiome homeostasis impairs enteric immunity, thereby increasing susceptibility to sepsis, a systemic inflammatory syndrome resulting from a dysregulated host response to infection, of gut microbial origin as well as elevating sepsis severity and mortality rates in older populations [96,97]. Notably, sepsis is both a driver and a consequence of gut microbiome disruption. During sepsis, profound shifts in gut microbiota are observed, including a precipitous loss of microbial diversity, expansion of opportunistic pathogens, and depletion of commensal bacteria [98]. These alterations arise from multiple interacting mechanisms, such as intestinal ischemia/reperfusion injury, systemic immune dysregulation, broad-spectrum antibiotic exposure, and altered nutritional intake during critical care, creating a self-perpetuating cycle in which dysbiosis amplifies sepsis pathogenesis through increased microbial translocation and heightened inflammatory responses, while sepsis further erodes microbiome stability and barrier integrity [97,99,100]. Our recent observational cohort study of eighteen sepsis patients revealed perturbations in entero-septic microbial metabolites affecting not only bacterial but also fungal communities, indicating pan-microbial dysbiosis that persists beyond the acute phase of sepsis [101,102].

Clinically, sepsis survivors, face heightened risks of morbidity, mortality, and long-term cognitive impairment [103,104], with the risk of dementia nearly doubling and persisting for over a decade following sepsis [105]. Moreover, patients with ADRD are highly vulnerable to immediate and sustained cognitive decline following infection-related hospitalizations [103,106]. Given that infection-induced sepsis affects millions worldwide each year, a substantial number of older adults may be at higher risk for premature cognitive decline during the dementia-prodromal phase. During sepsis, circulating pathogen-associated molecular patterns (PAMPs), such as LPS, and damage-associated molecular patterns (DAMPs) can cross the compromised BBB, triggering neuroinflammatory cascades characterized by microglial activation and elevated pro-inflammatory cytokines [107–109]. Dysbiosis exacerbates this process by increasing systemic PAMP and DAMP loads, thereby enhancing their translocation into the brain. Under normal conditions, *Candida*, a common opportunistic fungal genus in the gut, accounts for approximately 20-30% of the human gut mycobiome, but after sepsis, it can become overwhelmingly dominant, comprising up to 90–95% of the fungal population [102]. Notably, *Candida albicans*, one of the most increased species in sepsis patients, is well-known for causing candidemia and invasive candidiasis, highlighting the elevated risk of systemic fungal infections following sepsis. Furthermore, studies have shown that candidemia can lead to mild memory impairment that is reversible upon fungal clearance, suggesting a potential link between *Candida* infection and cognitive deficits [110,111].

Similarly, meningitis, a localized infection of the meninges that can progress to sepsis, triggers severe neuroinflammation, neuronal damage and irreparable disruptions to neural circuits, with survivors enduring long-term neurological sequelae including dementia [112]. For many meningitis cases, the site of infection initiation and mechanisms by which bacteria reach the brain remain unclear, but case studies have identified abscesses across physically distant organs and bloodstream infections [113]. Many pathogens involved in meningitis such as *K. pneumoniae* are gut inhabitants, implying that bacteria found in brain could originate from the gut and travel via systemic circulation [113,114], which may be a missing link between hospital-acquired infections, pathobiome, and increased AD risk, stressing the need to assess the role of such infectious gut–brain translocations in AD pathology. Supporting this, data from U.S. hospitals show a high rate of gut colonization by *K. pneumoniae* (45.4% of admissions), with patients exhibiting higher intestinal abundance being more likely to develop bacteremia, which is the presence of bacteria in the bloodstream, further suggesting a possible route for gut-derived bacteria to reach the brain [115]. Moreover, our recent findings in a transgenic AD mouse model show that specific opportunistic pathogens can translocate to the brain under dysbiotic conditions, directly inducing acute neuroinflammation and leading to neurobehavioral impairments [116].

In the context of ADRD, these inflammatory mechanisms are particularly concerning, as they may exacerbate AD pathology, including amyloid-beta accumulation [117]. Furthermore, systemic immune dysregulation and sustained peripheral inflammation following sepsis may drive chronic microglial overactivation, impair amyloid clearance, and promote long-term neuronal damage [118–120]. Thus, the relationship between sepsis and ADRD appears bidirectional: aging and ADRD-associated immune vulnerabilities increase susceptibility to severe sepsis, while the inflammatory and neurotoxic aftermath of sepsis may accelerate neurodegenerative processes—creating a vicious cycle that amplifies morbidity in older adults.

## Conclusions and prospects

Emerging evidence clearly suggests a strong link between gut microbiome and ADRD pathology and suggest that microbiome modulation might prevent/ameliorate this pathology, although this may involve pleiotropic mechanisms particularly given the intricate role of microbiome in multifarious epithelial, metabolic and inflammatory functions. In addition, aging-associated dysbiosis not only predisposes to chronic low-grade systemic inflammation but also increases vulnerability to acute inflammatory insults such as sepsis, a condition that itself can induce profound and persistent microbiome disruption. The bidirectional relationship between sepsis and ADRD underscores the need to integrate infection-related inflammatory events into the broader framework of ADRD risk and progression.

Despite these insights, much of the existing evidence drives from preclinical animal studies, while most clinical data are obtained from individuals already diagnosed with cognitive impairment, dementia or AD. These limitations extend to our own work and to many studies in this area, which have largely relied on pilot-scale or observational designs with relatively small sample sizes. Such constraints may limit statistical power and generalizability, necessitating cautious interpretation of the reported associations. Consequently, the precise cellular/molecular mechanisms underlying this intricate gut-brain interface in ADRD remains to be comprehended and are only beginning to be explicated, underscoring the need for further broader and systematic cross-sectional as well as prospective longitudinal studies to reveal progressive shifts in microbiome, metabolome, inflammasome, proteome and transcriptome in gut, blood and brain niches preceding and during the developmental stages of dementia and cognitive decline while controlling for potential confounding elements such as genetics, ethnicity, diet, lifestyle factors, medications, smoking/alcohol, etc.

Preclinical animal and cellular models are particularly valuable in this context, as they allow controlled induction of dysbiosis or sepsis-like states, and enable direct examination of how gut-derived microbes, endotoxins, and metabolites access the brain and interact with resident immune cells. These models also permit temporal resolution of disease processes, making it possible to distinguish whether microbiome alterations and sepsis-associated immune activation precede, exacerbate, or accelerate ADRD-related neuropathology. Importantly, such systems provide platforms for mechanistic validation of human observations, including testing whether specific gut pathobionts, microbial metabolites, or infection-induced inflammatory cascades can initiate or amplify neurodegenerative processes.

Such an integrative understanding may ultimately inform the development of novel microbiome-targeted therapies, including probiotics, prebiotics, fecal microbiota transplantation, and precision antimicrobials, particularly given the limited efficacy of amyloid- and tau-based approaches. Together, the existing evidence suggests that gut microbiome plays a pivotal role in host neurocognitive function, but further studies are needed to pin down specific microbiome species/strains and associated mechanisms/pathways underlying this gut–microbiome–brain axis in patients with cognitive impairment, dementia, and AD, which would help in discovering novel avenues for devising personalized preventive and therapeutic strategies.

**Summary points**

- Aging-related gut microbiome changes impair barrier function and promote chronic low-grade inflammation, increasing susceptibility to systemic infections and ADRD.
- Gut dysbiosis contributes to neuroinflammation via altered metabolites, impaired neurotransmitter balance, and microbial translocation.
- Sepsis both results from and worsens dysbiosis, triggering systemic inflammation and neuroinflammatory cascades that accelerate ADRD pathology.
- Future studies should identify key microbiome species and mechanisms in the gut–brain axis to guide personalized prevention and therapy for cognitive decline and AD.

## Open Access

Open access for this article was enabled by the participation of the University of Florida in an all-inclusive Read & Publish agreement with Portland Press and the Biochemical Society.

## Abbreviations

AD: Alzheimer’s disease
ADRD: Alzheimer’s disease and related dementias
BBB: blood–brain barrier
CNS: central nervous system
CSF: cerebrospinal fluid
DAMPs: damage-associated molecular patterns
LPS: lipopolysaccharides
MCI: mild cognitive impairment
PAMPs: pathogen-associated molecular patterns
TMAO: trimethylamine-N-oxide
